# Hospital In Home: Evaluating Need and Readiness for Implementation (HENRI) in the Department of Veterans Affairs: protocol for a mixed-methods evaluation and participatory implementation planning study

**DOI:** 10.1186/s43058-022-00338-7

**Published:** 2022-08-29

**Authors:** Jennifer L. Sullivan, Reza Yousefi-Nooraie, Derek D’Arcy, Adele Levine, Lindsey Zimmerman, Marlena H. Shin, Emily Franzosa, William Hung, Orna Intrator

**Affiliations:** 1 Center for Innovation in Long Term Service and Supports, Providence VA Healthcare System, Providence, USA; 2 Department of Health Services, Policy and Practice, Brown University School of Public Health, Providence, RI 02916 USA; 3 Department of Public Health Science, University of Rochester, Rochester, NY USA; 4 VA Finger Lakes Healthcare System, Canandaigua, NY USA; 5 Department of Health Law Policy and Management, Boston University School of Public Health, Boston, MA USA; 6 Office of Mental Health and Suicide Prevention, National Center for PTSD, Rockville, USA; 7 Stanford Department of Psychiatry and Behavioral Health, San Francisco, USA; 8 University of Washington Department of Psychiatry and Behavioral Health, Seattle, USA; 9 Center for Healthcare Organization and Implementation Research, VA Boston Healthcare System, Boston, MA USA; 10 Geriatric Research, Education and Clinical Center, James J. Peters VA Medical Center, Brookdale Department of Geriatrics and Palliative Medicine, Icahn School of Medicine at Mount Sinai, New York, USA

**Keywords:** Hospital at Home, Veterans, Home care, Older adults, Innovation

## Abstract

**Background and objectives:**

The Department of Veterans Affairs (VA) Hospital-In-Home (HIH) program delivers patient-centered, acute-level hospital care at home. Compared to inpatient care, HIH has demonstrated improved patient safety, effectiveness, and patient and caregiver satisfaction. The VA Office of Geriatrics & Extended Care (GEC) has supported the development of 12 HIH program sites nationally, yet adoption in VA remains modest, and questions remain regarding optimal implementation practices to extend reach and adaptability of this innovation. Guided by theoretical and procedural implementation science frameworks, this study aims to systematically gather evidence from the 12 HIH programs and to develop a participatory approach to engage stakeholders, assess readiness, and develop/adapt implementation strategies and evaluation metrics.

**Research design and methods:**

We propose a multi-phase concurrent triangulation design comprising of (1) qualitative interviews with key informants and document review, (2) quantitative evaluation of effectiveness outcomes, and (3) mixed-methods synthesis and adaptation of a Reach Effectiveness Adoption Implementation Maintenance (RE-AIM)-guided conceptual framework.

**Results:**

The prospective phase will involve a participatory process of identifying stakeholders (leadership, HIH staff, veterans, and caregivers), engaging in planning meetings informed by implementation mapping, and developing implementation logic models and blueprints. The process will be assessed using a mixed-methods approach through participant observation and document review.

**Discussion and implication:**

This study will support the continued spread of HIH programs, generate a catalog of HIH implementation evidence, and create implementation tools and infrastructure for future HIH development. The multi-phase nature of informing prospective planning with retrospective analysis is consistent with the Learning Health System framework.

**Supplementary Information:**

The online version contains supplementary material available at 10.1186/s43058-022-00338-7.

Contributions to the literature

- Older adults are vulnerable to complications and adverse events of inpatient hospitalization; home hospital care can improve patient safety and outcomes and aligns with patient and caregiver preferences. The VA has pioneered the Hospital In Home model, but sustainable spread requires an in-depth understanding of the implementation processes and readiness for change.
- This protocol describes a mixed-methods evaluation of implementation determinants and effectiveness to inform the development of a readiness assessment survey and logic models and implementation blueprints for HIH implementation at new sites.
- Findings will be used to develop an implementation guidebook to assist in the implementation planning efforts of this care delivery innovation.

## Reporting standards

All contributions to the design, conduct, interpretation, and reporting of this study will be recognized through the authorship of the resulting reports and peer-reviewed publications. Any adverse events will be reported to VA Central IRB.

## Background

The population is aging rapidly in the USA; the population aged 65 and above was estimated to be 43.1 million in 2012 but is projected to double to 83.7 million in 2050 [1]. Older adults experience higher levels of chronic disease, with more than 60% living with 2 or more chronic conditions [2] and 17% with 4 or more chronic conditions [3], and often take multiple medications to manage these illnesses. Older adults also disproportionately account for more hospital admissions and admission days [4] and are more at risk for adverse events and complications during acute care hospitalization [5]. Recent data indicate that 25–50% of hospitalized older adults experience delirium [6], 3–15% experience pressure ulcers [7], and 4.7% experience hospital-related infections [8], e.g., catheter-associated infections. Adverse events and complications may ultimately lead to functional decline that requires institutionalization [9–11]. Older patients, particularly those who have been hospitalized before, may prefer treatment at home rather than in the hospital [12, 13]. For these reasons, a novel model of care was developed in the 1990s in the USA to deliver acute care to older adults who may otherwise require hospital admission [14].

In the Hospital at Home (HAH) model, patients’ needs are met in an environment where hazards and adverse events are less likely to occur [15]. Through HAH, patients with acute medical conditions that meet the inpatient admission criteria and who traditionally require hospital treatment for illnesses such as pneumonia, cellulitis, heart failure, and other acute care conditions can be treated at home via clinician visits, medication administration including intravenous medicines, and tests such as X-rays, laboratory test, and electrocardiograms [16]. This trial included 3 sites, one of which is a site in the Veteran Health Administration (VA) [17]. The feasibility and safety of HAH care, the community equivalent of Hospital in Home (HiH) in the VA, have been previously demonstrated; meta-analysis with multiple trials has shown that HAH was associated with reduced mortality (21% relative risk reduction with a number needed to treat of 50), and reduced hospital readmission (RRR 24%) [18]. Patient satisfaction is also superior to hospital care [15, 19], and HAH care is associated with reduced caregiver stress [20]. When compared to inpatient hospital stay, costs associated with HAH were lower (average 19% savings per hospitalization episode) [21]. HAH had a 74% reduction in the risk of delirium during the acute care episode with an associated reduction in the incidence of other adverse events [22]. Fewer HAH patients need nursing home care after hospitalization, and functional outcomes were improved such that older adults could maintain their independence at home after discharge [23].

Because the HAH model has the potential to avoid this cascade of events detrimental to patients’ health and support functional independence for older adults, VA began piloting a limited number of additional HiH programs in 2010. Despite the evidence of its benefits to patient care and to health systems, the HIH model has not been widely adopted due to financial and regulatory barriers, and even with adoption in different sites, there are variations in program setups and in reach, adoption, implementation, and potentially effectiveness. To duplicate the functions of acute care-level hospital, HIH programs need to adapt clinical practice protocols for medication administration, laboratory, and other testing; identify and use durable medical equipment; establish clinical teams; adapt clinical documentation; and ensure seamless communication and transfer of care protocols. The VA has been a leader in adopting HIH supported through transformative initiatives (T21) by the VA Office of Geriatrics and Extended Care (GEC) since the early 2010s [24]. Comparative effectiveness evaluations reported HIH was associated with reduced costs, lower likelihood of nursing home placement, with no impact on mortality or re-hospitalizations [25–27], yet preliminary data on 7 VA HIH programs showed substantial variation in program setup, staffing, referral processes, targeted populations, and services offered between programs.

This lack of documentation of implementation barriers and facilitators limits further uptake by other Veterans Affairs Medical Centers [28]. Estimates using Medicare data from 2015 found that 20% of hospital admissions were eligible for HAH participation, suggesting a potential cost savings of $144.6M to Medicare [29] and $85M in direct costs to the VA per year.

## Overview of HENRI

**Fig. 1:**
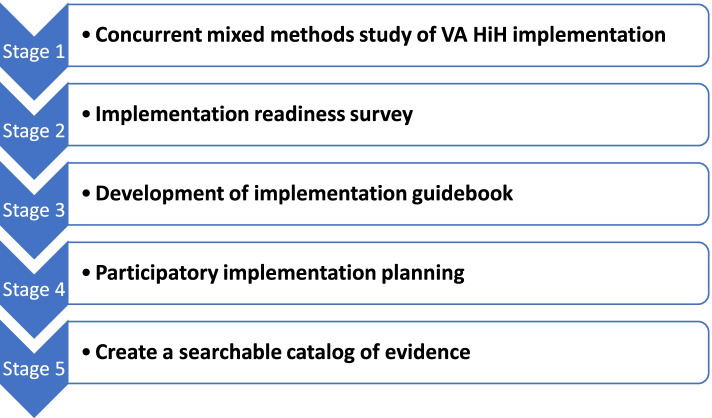
HENRI study design

Given increased interest and need for this innovative model of care, we developed the Hospital In Home: Evaluating Need and Readiness for Implementation (HENRI) study to fill the current gap in HIH implementation knowledge using a multi-stage approach. Figure 1 displays the stages of the study. HENRI involves:

- Stage 1: a mixed-methods analysis of 12 existing VA HIH programs (informed by the Reach, Effectiveness, Adoption, Implementation, Maintenance (RE-AIM) [30] and Practical, Robust Implementation and Sustainability Model (PRISM) [31]) [32]
- Stage 2: assessment of readiness to implement HIH across VA Medical Centers (guided by the Theory of Organizational Readiness for Change [33]).
- Stage 3: development of implementation guidebook compiling implementation strategies, tools, and processes of care
- Stage 4: a participatory process of engaging local stakeholders from 10 new VA sites (with the greatest evidence of readiness) in planning for future implementation of HIH through the development of implementation logic models and blueprints, guided by Implementation Mapping and System Science approaches
- Stage 5: creating a searchable catalog of evidence

Through these innovative multi-stage processes, HENRI will further a growing evidence base and generate a suite of implementation tools using data from 12 VA HIH programs. The results will inform implementation planning and adaptation thus increasing the probability of successful adoption of HIH and its sustainment and growth. We have received approval from the VA Central Institutional Review Board (IRB) for all study procedures.

The objective of this study is to systematically gather evidence from the 12 HIH programs and to develop a participatory approach to engage stakeholders, assess readiness, and develop/adapt implementation strategies and evaluation metrics.

## Setting

The VA has made investments in establishing home-based primary care programs in each of its VA Medical Centers, thereby establishing teams and expertise with in-home care, which serves as a base to build HIH programs in many sites. The environment in VHA, with its single integrated health care system that bridges inpatient and outpatient care, may facilitate this growth. Challenges encountered outside of the VA such as payment mechanisms may be different in VA. An early adopter of electronic health record systems, each VA medical center has clinical informatics staff that has the capability of adapting records to support clinical innovations. VA has fostered innovative approaches for older adults through innovative care models supported through transformative initiatives (T21) by the VA Office of GEC since the early 2010s. These provided both technical and funding support for field teams to develop, test, and adopt potentially effective care models. HIH programs have been supported and disseminated through these mechanisms starting from a single program to 12 programs in 2019.

## Stage 1: Concurrent mixed methods study of HIH implementation in VA

The aim of stage 1 is to establish evidence regarding how the HiH program was implemented at the 12 existing VA HIH sites using mixed methods.

### Qualitative phase

#### HIH staff sampling and recruitment

We will obtain the names of the HH Director, HiH team members, and leadership from the HIH director after a site has consented to participate in the study. We will recruit up to 120 HIH staff (10 per site, as typical sample sizes for implementation studies in hospitals ranges between 5 and 10 individuals for interviews) [34, 35]. The VA National Director of HIH will approach sites to collect contact information for potentially interested staff from each site’s HIH point of contact. VA employees who are HIH key informants, including HIH staff, front-line clinical staff interfacing with HIH program, managers, and senior leaders will be invited to participate. One hundred twenty participants will be recruited for qualitative interviews.

#### Patients and caregiver sampling and recruitment

In addition, we will interview and recruit up to 35 patients and 35 caregivers from participating HIH sites.

### Data collection

#### HIH staff

Interviews will focus on the components of RE-AIM and PRISM contextual elements. We will conduct interviews using a semi-structured interview guide via phone with the study staff on RE-AIM outcomes [30, 32] to gain an in-depth understanding of the contextual factors influencing HIH implementation. We will also ask the medical director of the HIH for documentation or policies they have, which will allow us to understand the changes to the program over time.

#### Patient and caregiver

Veteran interviews focus on experiences regarding program participation, satisfaction, and suggestions for improvement. In addition to these focal areas, care partner interviews also assess caregiver burden. Those who agree to participate in the study will complete the informed consent procedures and will be scheduled for a brief semi-structured qualitative interview. Veterans and their caregivers will be compensated $20 upon completion of the interviews.

#### Data analysis

All interviews will be audio-recorded and professionally transcribed. Using NVivo 12, the qualitative coding team will review and independently the code transcripts. A priori constructs, consistent with the RE-AIM-PRISM framework [36] will be coded first. As coding proceeds, based on team discussions, new coding categories will be identified, elaborated, and expanded in an iterative fashion to capture emergent themes [37]. The list of codes will eventually evolve to a point where the existing categories are sufficient to cover all new interview material being processed. When no new concepts are discovered in the interview notes, a condition of “saturation” will be achieved [35, 38, 39]. Inter-rater reliability will be established using the “check-coding” process where both coders will independently code the same interview notes, and reliability estimates between all pairs of coders will be computed. Coders will then meet to compare their scoring, discuss areas of disagreement, and continue coding to achieve full agreement.

Upon completion of coding, we will run domain reports by RE-AIM-PRISM dimensions for each site. The components of RE-AIM will be captured using a structured within-site matrix containing consolidated and summarized qualitative data for each site as well as exemplar quotes. The PRISM contextual components [31] will be summarized at the site level using within-site case summaries of the data for each PRISM component and exemplar quotes. Upon completion of analysis at the 12 individual sites, data will be combined into a cross-site matrix to descriptively assess the similarities and differences across the sites. The resulting cross-site matrix will be used to assess the differences by RE-AIM performance [37].

### Quantitative phase

#### Data collection

We propose a retrospective review of VA Corporate Data Warehouse (CDW) to retrieve data on RE-AIM Dimensions from all Veterans receiving care in HIH between fiscal years 2014–2019, identifying subjects based on outpatient visits with primary or secondary stop code 354. The primary and secondary stop codes refer to the main clinical groups or units where a patient received care during their visit. We will then retrieve data on Veterans who received care in the hospital during the same years and in the same parent VA Medical Center. All data will be securely stored on VA Informatics Computing Infrastructure (VINCI) to assure confidentiality.

#### Analysis

We will summarize the characteristics of veterans receiving HIH care in the 12 programs including socio-demographics/enrollment (e.g., age, race, marital status, priority status, Medicare enrollment, Medicare-Medicaid dual enrollment), chronic conditions, and other health conditions and risk measures at the time of HIH event including Care Assessment of Need [40], Centers for Medicare and Medicaid Services (CMS) hierarchical condition categories (HCC) score and categories [41], JEN Frailty Index (which predicts the probability of long-term institutionalization [40–43]), and the VA’s Nosos risk adjustment score predicting costs (i.e., based on CMS HCC scores and other VA-specific data including mental health care, pharmacy, patient demographics, and VA costs) [43, 44]. Health care utilization prior to index stay will be summarized including inpatient, emergency department, and nursing home use 30 and 180 days prior to HIH admission. For an early discharge model, we will also summarize the characteristics of the hospital stay (e.g., intensive care unit use, DRG, length of stay). Health care costs during 30 and 90 days after discharge and costs of the index event will be compared. Characteristics will be tested for inclusion as confounders in the propensity score model.

As the existing programs did not incorporate a comparison group in the design, we will use available administrative data to construct an artificial comparison group using propensity score methods. Specifically, we will obtain the primary diagnosis and diagnostic-related groups (DRGs) (for enrollees in the early discharge model) and find non-enrolled veterans who had the same primary diagnosis and/or DRG. We will study the effect of the program in its first and last 2–3 years of implementation (as available) by selecting patients receiving HIH only during those time periods. This will allow us to also evaluate changes in effectiveness due to program maturation. To apply propensity score analysis, we will first estimate the probability of being treated in HIH among veterans receiving HIH care and potential comparisons based on the identified covariates using logistic regression. The potentially comparable veterans will then be matched to program enrollees based on the estimated probability of receiving the services. As a comparative effectiveness analysis, we will estimate the average treatment effect on the treated (ATT), which captures the average differences in outcomes between HIH patients and matched veterans [25, 45–47].

A simple power calculation shows that 879 matched HIH, and comparisons are needed to identify an effect size of 0.2 with 90% power at 5% significance for a 1-sided test with 8 sites with an intra-class correlation of 0.49. This sample size can be achieved with about 100–150 veterans who received care in HIH per site.

### Mixed-methods integration

Table 1 displays the RE-AIM dimensions and example concepts, source materials, and measures. Qualitative RE-AIM results and quantitative RE-AIM data will be assembled into a matrix (see Supplemental Material). We will use a team consensus process to rate the level of success across each RE-AIM dimension on a 5-point scale; once RE-AIM matrices are complete for each site, we will present the rating to our Advisory Committee. Based on this feedback, we will recalibrate as necessary and divide sites into three RE-AIM groups: high, medium, and low performance.

**Table 1:** RE-AIM dimensions and example concepts, source material, and measures

REAIM dimension and concepts	Source material	Instrument or measurement
**Reach**		
Inclusion/exclusion criteria	Team interviews; metrics	# screened; % approached; % excluded (clinical; geographic; others)
Individuals who participate, based on a denominator	Collected by the local team; denominator (local team definition)	% served
Characteristics of HIH patients	Office of Geriatrics and Extended Care Data Analysis Center (GECDAC) data	Age, sex, race/ethnicity, marital status, priority status, admission diagnosis, complexity (e.g., comorbidities, Jen Frailty Index, hierarchical condition categories, Nosos score, high needs high risk, prior utilization)
Patient recruitment	Team interviews	Steps utilized by teams to recruit/reach patients
**Effectiveness**		
Overall effect of HIH	GECDAC data; HIH/propensity score-matched comparison	Outcomes: mortality, 30/90 day readmission, length of stay, hospital/HIH cost
Characteristics of HIH patients	GECDAC data	Same as used for reach
Variations in effectiveness	Team interviews (exploratory)	Identify barriers, facilitators, potential reasons for variations in effectiveness
Veteran and caregiver satisfaction; caregiver burden	Veterans and caregiver interviews	How satisfied are you with (your/your loved one’s) HH care? 5-point Likert
**Adoption**		
Level of staff adoption	Stakeholder/team interview	HiH staff adoption of the HiH model, awareness, and receptivity referring staff are to the HiH program
Patient drop out	Stakeholder/team interview	% patients dropped out of HiH
Vendor/contractor participation	Team interviews	# of vendors; types of vendors; issues/challenges, encountered with vendors
**Implementation**		
Adherence to program guidelines	Team interviews; metrics from programs	Summary score 5 items (items on a 0–2 scale): staff training and competencies; clinical documentation; communication; clinical standards; quality indicators
Adaptations made	Team interviews	Identify adaptations made, reasons why
Start-up cost of the program	Team interviews/GECDAC data follow-up	Administrative costs to setup positions/contracts; startup equipment cost/training cost
**Maintenance**		
Program growth	Team interviews, GECDAC data	Changes in inclusion/exclusion criteria; # of Veterans/ month; # visits/month
Integration into routine practices and policies	Team and leadership interviews; local policy documents	Were any electronic medical record patches/databases created for the program? Any positions permanently allocated to the program?

Once RE-AIM performance status has been assigned to each site, we will explore the facilitators and barriers at sites in each of the 3 RE-AIM categories. We will focus on PRISM contextual elements because we are interested in understanding which of the contextual elements and implementation practices lead to the full implementation of HIH. These analyses will be conducted using a cross-site matrix approach where we will review each grouping of sites (high, medium, and low). We will review quotes for each PRISM element and rate the influence on implementation as “positive,” “negative,” or “mixed” (i.e., the site has both positive and negative evidence). We will then compare these ratings across the sites in the performance category and between performance categories. These methods have successfully been implemented in a previous VA study [48]. For example, we may find that sites in the higher-performing RE-AIM group had more leadership support and better networks and communication than lower- or middle-performing sites. As needed, coders will refer to coded transcript data and quantitative findings to further contextualize results. These data will inform the development of implementation tools.

## Stage 2: Implementation readiness survey

In stage 2 of the study, we assess the organizational readiness to change which will allow us to identify new 20–30 VA Medical Centers most ready and motivated to implement the HIH program.

### Survey adaptation

To assess the readiness of organizational change, we will refine and develop an online survey to assess the readiness of prospective sites for HIH guided by the PRISM perspective with additional questions motivated by the Theory of Organizational Readiness for Change [33]. This theory proposes that organizational readiness is defined based on two constructs of change: commitment (organizational members’ shared resolve to pursue the implementation), and change efficacy (organizational members’ shared beliefs in their collective capabilities to organize and execute implementation), which together influence implementation effectiveness. Several readiness assessments have been developed to address this multi-dimensional construct. For example, the Organizational Readiness to Change Assessment (ORCA) is a 77-item instrument that was developed and tested in the context of the Veterans Health Administration [49, 50]. Gagnon et. al. (2014) conducted a systematic review and identified 26 eligible instruments measuring readiness for change [49]; we will adapt and refine the most relevant instruments from this review to develop a set readiness assessment survey relevant to the implementation of HIH at various decision-making levels. The final survey will include items to address the readiness of organizational change as well as specific site needs and readiness to adopt HIH as identified in stage 1.

We will perform pilot testing of survey items with at least two interdisciplinary staff members (physicians, nurse practitioners, nurse, etc.) from two current sites of HIH and two prospective interested sites for HIH adoption to assess ease of administration, respondent burden, and clarity. Formal post-survey interviews with cognitive interview techniques will be conducted to assess whether items are understood as intended, to determine whether items are redundant, and to identify cognitive processes involved in answering the questions [51, 52]. Suggestions will also be solicited from survey respondents for additional items to be vetted by the expert and stakeholder team.

### Survey administration

#### Sampling and recruitment

Twenty to thirty prospective sites for readiness survey administration will be recruited based on the HIH call for proposals. Engagement with the site staff will be facilitated by GEC leadership. After consultation with the main contact and leadership at each prospective site, a list of stakeholders at various levels of decision-making (front-line staff, administration, organizational leaders) will be identified and invited to participate in the readiness survey. We expect about 20–30 sites interested in responding to the survey. The scores will be summarized based on the domains of the Theory of Organizational Readiness for Change by Weiner (2009) [33] and will be reported as mean (standard deviation), for each prospective site.

## Stage 3: Development of implementation guidebook

The aim of stage 3 is to create an implementation guidebook informed by stage 1–2 findings which will inform new VA Medical Centers starting HIH programs about implementation approaches they can adopt at their own site.

We will assemble tools and procedures from each of the existing HIH programs based on the qualitative interviews conducted. These tools will cover the implementation of the HIH model at various stages of implementation including the pre-implementation stage: obtaining buy-in from leadership; identifying and obtaining resources for support of program; building a team for the program through postings for recruitment of needed staff, staff training protocols, and credentialing procedures, contracts with vendors, standard operating procedures for service delivery, medication administration and other aspects, and tools for screening; and identifying and consenting veterans and caregivers to receive HIH care, quality assurance tools, and metrics and surveys used for tracking outcomes. The items will be examined for the similarities, and adaptations will be noted. A summary guidebook will be assembled, and items will be compiled. These tools will be reviewed by subject matter experts on the Advisory Committee for comprehensiveness, clarity, and relevance. Concrete steps to implementation will be included in the guidebook to assist new sites. Based on stage 1 findings, and the results of the readiness survey, we will summarize the list of implementation barriers in general and in specific prospective sites*.*

## Stage 4: Participatory implementation planning

In stage 4, we will engage 10 of the 20–30 VA sites that participated in stage 2 and who are the most ready and motivated to implement the HiH program, to participate in a participatory pre-implementation planning activity to prepare for future HIH program implementation.

Implementation mapping, a recent adaptation of intervention mapping [53] to the context of D&I, is a novel participatory approach of engaging stakeholders in systematically developing and adapting implementation strategies [54]. Participatory system dynamics involves group model building (GMB) as a collaborative learning process engaging stakeholders in depicting the complexity of the dynamics of the systems. GMB workshops are scripted approaches that run similar to a focus group and provide rich, standardized frameworks to describe systems [55, 56]. This approach helps stakeholders and researchers learn, compare, and communicate about causal mechanisms and potential solutions to improve measurable system-level outcomes. The unique advantage of system dynamics methods is the possibility of developing causal loop diagrams to depict the non-linearity and complexity of interactions between various factors, and the possibility of incorporating these diagrams in mathematical simulations. The structure of the sessions will be adapted from the implementation mapping model and scripts for running GMB workshops [56].

### Sampling and recruitment

Ten sites considering implementation of a HIH program that participated in the readiness survey will be engaged. At each site, participants with various decision-making and practice levels will be invited to the focus group sessions.

#### Implementation planning focus groups

The participants from 10 prospective VA Medical Centers will be invited to participatory sessions to develop implementation logic models and implementation blueprints. Focus group discussions will focus on planning for implementation strategies and evaluation frameworks, which are adapted from implementation mapping procedures. The session structure will be adapted from GMB “action ideas” script [56].

Participants identify variables and causal relationships between variables that influence the system following instructions on feedback mechanisms and non-linearity. After the meeting, the researchers will develop a one-page implementation logic model along with the causal loop diagram [57] to summarize the discussions in planning meetings. The logic model and the diagram will be sent to the stakeholders for comments. The focus of the current proposal is using these diagrams as schematic presentations of the complexity of the systems and the role of action points/interventions, to provide qualitative insight to participants. The transcripts of the sessions and the resulting logic models and causal loop diagrams will be analyzed thematically across sites, to provide an overarching insight into the process of stakeholder engagement in planning and common and site-specific goals, mechanisms, and challenges.

## Stage 5: Creating a searchable catalog of evidence

In the final stage of our study, we compile the results and themes from stages 1–4 into a database to allow any VA site interested in implementing the HIH Program (including those with existing HIH programs, those participating in stages 2–4, and new VA Medical Centers) to search for results which are most applicable to them as they prepare for implementation.

We will perform a content analysis of project documents (meeting notes, evaluation reports of HIH sites, summary reports of implementation activities, and publications) using a thematic analysis framework [58]. The analysis will be done using framework approach [59] which is a quick and applied qualitative analysis model to facilitate and speed up the analysis when a priori frameworks are available. The guiding conceptual frameworks will be RE-AIM, PRISM, and the main themes of local implementation of HIH. The resulting themes will be stored as searchable keywords on an online platform. SharePoint will be used as a vehicle for this repository. The Sharepoint will be designed to allow the project staff to upload one or more documents per record, and group or filter based on the fields we include in the list. Fields will minimally include date created, date uploaded, type of document (transcript, email, abstract, etc.), keywords, creator, and an indicator of whether it should be included in reports.

## Discussion

The study protocol detailed in this manuscript involves close partnership with VA and GEC leadership to identify implementation determinants and triangulate these data to create an actionable HIH readiness assessment and implementation tools. The multi-phase study design, guided by the RE-AIM-PRISM framework, will generate evidence of quantitative effectiveness and qualitative implementation outcomes and innovate a newer scientific methodology—implementation mapping, use of logic models, and blueprints, and the creation of a searchable catalog of results that will be useful beyond the context of HIH implementation.

Findings from all phases will be integrated into an implementation guidebook to inform future HIH implementation efforts within the VA and HAH efforts in other delivery systems. Older adults may benefit from efforts to divert hospital care to home settings. Over the past decade, the VA has pioneered Hospital In Home programs that are associated with improved patient safety, satisfaction, and cost savings. We anticipate that our systematic and adaptive approach to evaluate and scale up HiH will be more successful in recognizing and addressing the complexities of implementation and sustainment of HIH, as a complex intervention [60, 61].

This study has several implications to move the science of D&I forward. Two frameworks of R-EAIM and PRISM have been recently integrated into a more comprehensive model to further capture the complexities of implementation [32]. Our approach to the joint application of both frameworks will inform their points of interface and missing links and will also provide evidence to the impact of this integration. Our approach to merge implementation mapping and Group Model Building, two implementation planning approaches that are rooted in different disciplines, will provide empirical evidence to best develop best practices to engage stakeholders in planning for the implementation of complex interventions.

## Supplementary Information

**Additional file 1: Table S1.** Method to synthesize RE-AIM dimension ratings.

## Data Availability

Not applicable, as this manuscript does not contain any data.

## Abbreviations

ATT: Average treatment effect on the treated
CAN: Care Assessment of Need
CMS: Centers for Medicare and Medicaid Services
D&I: Dissemination and implementation
DRG: Diagnostic-related group
EMR: Electronic medical record
GEC: Office of Geriatrics & Extended Care
GECDAC: Office of Geritrics and Extended Care Data Analysis Center
HENRI: Hospital In Home: Evaluating Need and Readiness for Implementation
HAH: Hospital at Home
HCC: Hierarchical condition categories
HIH: Hospital in Home
JCAHO: Joint Commission Accreditation of Healthcare Organizations
OCC: Office of Connected Care
OCS: Office of Caregiver Support
ORCA: Organizational Readiness to Change Assessment
PRISM: Practical, Robust Implementation and Sustainability Model
PTAC: Physician-Focused Payment Model Technical Advisory Committee
RE-AIM: Reach, Effectiveness, Adoption, Implementation, Maintenance
VA: Department of Veterans Affairs
VINCI: VA Informatics Computing Infrastructure
VIReC: VA Information Resource Center
