# Supplementary material for: Hospital In Home: Evaluating Need and Readiness for Implementation (HENRI) in the Department of Veterans Affairs: protocol for a mixed-methods evaluation and participatory implementation planning study
Source: Implement Sci Commun. 2022 Aug 29;3:93. doi: 10.1186/s43058-022-00338-7 (PMC9422109; doi:10.1186/s43058-022-00338-7)
Supplement: Supplementary file 1 — Additional file 1: Table S1. Method to synthesize RE-AIM dimension ratings. [file 43058_2022_338_MOESM1_ESM.docx]

**Supplemental Material**

**SM1. Method to synthesize RE-AIM dimension ratings**

| **[RE-AIM Dimension and Concepts** | **Method to Synthesize Data** |
| --- | --- |
| **Reach** | |
| Inclusion/ Exclusion criteria | Review all concepts. Based on findings, rate sites on a 5-point scale.  Anchors:  0 = no criteria met  1 = somewhat met  2 = about half met  3 = mostly met  4 = all met |
| Individuals who participate, based on denominator |  |
| Characteristics of participants |  |
| Patient Recruitment |  |
| **Effectiveness** | |
| Overall effect of HIH | Rate each of 5 HIH outcomes (0= no effect, 1=effect)  Summarize the # dimensions effective (0-5)  Review all concepts (e.g., satisfaction, burden, variations in effectiveness) in addition to overall effect. Rate using the following scale.  Anchors:  0 = no criteria met  1 = somewhat met  2 = about half met  3 = mostly met  4 = all met |
| Variations in effectiveness |  |
| Veteran and caregiver satisfaction; caregiver burden |  |
| **Adoption** | |
| Level of staff participation/adoption | Rate through group consensus using the following scale:  Anchors:  0 = no criteria met  1 = somewhat met  2 = about half met  3 = mostly met  4 = all met |
| Patient drop out |  |
| Vendor/ contractor participation |  |
| **Implementation** | |
| Adherence to program guidelines | Initial elements will be scores from 0-2 (not present to fully present). We will then rescore.  Anchors:  0 = no criteria met  1 = somewhat met  2 = about half met  3 = mostly met  4 = all met  *Data on adaptions and start-up costs supplemental to adherence rating. |
| Adaptations made* |  |
| Start-up cost of program* |  |
| **Maintenance**. | |
| Program growth | Rating of likelihood that program continued will be sustained.  Anchors:  0 = unlikely (no plans)  1 = somewhat unlikely  2 = could go either way  3 = likely  4 = very likely  *Data on length of time the program has been implemented is supplemental to maintenance rating.] |
| Integration into routine practices and policies |  |
